# Ingesting a pre-workout supplement containing caffeine, B-vitamins, amino acids, creatine, and beta-alanine before exercise delays fatigue while improving reaction time and muscular endurance

**DOI:** 10.1186/1743-7075-9-28

**Published:** 2012-03-30

**Authors:** Brandon D Spradley, Kristy R Crowley, Chih-Yin Tai, Kristina L Kendall, David H Fukuda, Enrico N Esposito, Sarah E Moon, Jordan R Moon

**Affiliations:** 1 Department of Sports Fitness and Health, Human performance and body composition laboratory, United States Sports Academy, 1 Academy Drive, Daphne, AL 36526, USA; 2 Department of Health and Exercise Science, Metabolic and body composition laboratory, University of Oklahoma, Norman, OK, USA; 3 University of South Alabama, College of Medicine, Mobile, AL, USA

**Keywords:** Branch-chained amino acids, Recreationally-trained, Human performance

## Abstract

**Background:**

The purpose of this study was to determine the effects of the pre-workout supplement Assault™ (MusclePharm, Denver, CO, USA) on upper and lower body muscular endurance, aerobic and anaerobic capacity, and choice reaction time in recreationally-trained males. Subjective feelings of energy, fatigue, alertness, and focus were measured to examine associations between psychological factors and human performance.

**Methods:**

Twelve recreationally-trained males participated in a 3-week investigation (mean +/- SD, age: 28 +/- 5 y, height: 178 +/- 9 cm, weight: 79.2 +/- 15.7 kg, VO_2max_: 45.7 +/- 7.6 ml/kg/min). Subjects reported to the human performance laboratory on three separate occasions. All participants completed a baseline/familiarization day of testing that included a maximal graded exercise test for the determination of aerobic capacity (VO_2max_), one-rep maximum (1-RM) for bench and leg press to determine 75% of 1-RM, choice reaction tests, and intermittent critical velocity familiarization. Choice reaction tests included the following: single-step audio and visual, one-tower stationary protocol, two-tower lateral protocol, three-tower multi-directional protocol, and three-tower multi-directional protocol with martial arts sticks. Subjects were randomly assigned to ingest either the supplement (SUP) or the placebo (PL) during Visit 2. Subjects were provided with the cross-over treatment on the last testing visit. Testing occurred 20 min following ingestion of both treatments.

**Results:**

Significant (*p *< 0.05) main effects for the SUP were observed for leg press (SUP: 13 ± 6 reps, PL: 11 ± 3 reps), perceived energy (SUP: 3.4 ± 0.9, PL: 3.1 ± 0.8), alertness (SUP: 4.0 ± 0.7, PL: 3.5 ± 0.8), focus (SUP: 4.1 ± 0.6, PL: 3.5 ± 0.8), choice reaction audio single-step (SUP: 0.92 ± 0.10 s, PL: 0.97 ± 0.11 s), choice reaction multi-direction 15 s (SUP: 1.07 ± 0.12 s, PL: 1.13 ± 0.14 s), and multi-direction for 30 s (SUP: 1.10 ± 0.11 s, PL: 1.14 ± 0.13 s).

**Conclusions:**

Ingesting the SUP before exercise significantly improved agility choice reaction performance and lower body muscular endurance, while increasing perceived energy and reducing subjective fatigue. These findings suggest that the SUP may delay fatigue during strenuous exercise.

## Background

Pre-workout supplementation has become a fundamental component in nutrition programs and a growing interest in the sports nutrition industry. Recent research indicates energy drinks are the most popular supplement next to multi-vitamins in the young adult population (18-35 year) [1]. Many athletes believe supplementation prior to training will result in greater focus, quicker reaction time, and increased power [1]. Performance-enhancing claims of dietary supplements have not been fully addressed in the context of sport-specific exercises. Therefore, examining the effects of nutritional supplements during an exercise training session has the potential to elucidate more practical recommendations and applications [2].

Most supplements contain different ingredients to produce ergogenic effects. When ingested together, these ingredients may work synergistically to enhance various aspects of exercise performance. The ergogenic effects of caffeine have been attributed to a number of possible mechanisms, primarily the blocking of adenosine receptors [3,4]. Caffeine inhibits action at adenosine receptors, which has been reported to decrease the perception of pain and effort, resulting in improved exercise performance [5,6]. Research has also suggested that adenosine-receptor antagonism contributes to improved performance via increases in neurotransmitter release and motor firing rate [3,7]. However, the effects of caffeine in combination with beta-alanine, branched chain amino acids (BCAAs), creatine, vitamin B-6, and vitamin B-12 on ratings of perceived effort and pain have not been well investigated, particularly when ingested as a pre-workout supplement. Both beta-alanine and creatine have been shown to delay the onset of neuromuscular fatigue and therefore potentially augment the ergogenic effect of caffeine [8,9]. Research has shown vitamin B-6 to play an important role in the metabolic pathways required for exercise [10], while vitamin B-12 assists with DNA synthesis, which is necessary for the formation of red blood cells [10]. Research suggests that when supplementing with BCAAs prior to physical activity, recovery improves due to an increase in protein synthesis and a reduction in protein degradation [11]. However, studies have indicated that BCAAs do not improve endurance performance [12]; therefore, further research is required to examine the effects of BCAA supplementation on various team sport-related performances (e.g. repeated sprint ability) [3].

Few studies have examined the effect of pre-workout supplements using protocols related to sports-specific training and team sport performance. Therefore, the purpose of this study was to determine the effects of a supplement containing BCAAs, creatine, beta-alanine, citruline malate, arginine, vitamin B-6, vitamin B-12, and caffeine (Assault™, MusclePharm, Inc., Denver, CO, USA) on upper and lower body muscular endurance, aerobic and anaerobic capacity, and choice reaction time.

## Methods

### Participants

Twelve recreationally-trained males participated in the three-week investigation (mean +/- SD, age: 28 +/- 5 y, height: 178 +/- 9 cm, weight: 79.15 +/- 15.7 kg, VO_2max_: 45.7 +/- 7.6 ml/kg/min). The investigation was approved by an Institutional Review Board for use of human subjects, and all subjects signed an informed consent and completed a health history questionnaire prior to the investigation. All subjects met the necessary inclusion requirements as assessed by a health history and exercise questionnaire. Subjects were required to have been free of any nutritional supplements or ergogenic aids for at least 6 weeks preceding the study and were asked to refrain from using any additional supplements during the course of the study. In addition, subjects regularly engaged in exercise activity on a consistent basis (workout days per week: 3.9 +/- 1.3 days, workout hours per day: 1.7 +/- 1 h, cardio training per week: 3.8 +/-1.8 h, resistance training per week: 4.3 +/- 1.8 h). Subjects also reported participation in one or more of the following sport activities: running, weightlifting, swimming, cycling, tennis, baseball, basketball, football, kickboxing, lacrosse, and mixed martial arts.

### Study design

**Figure 1:**
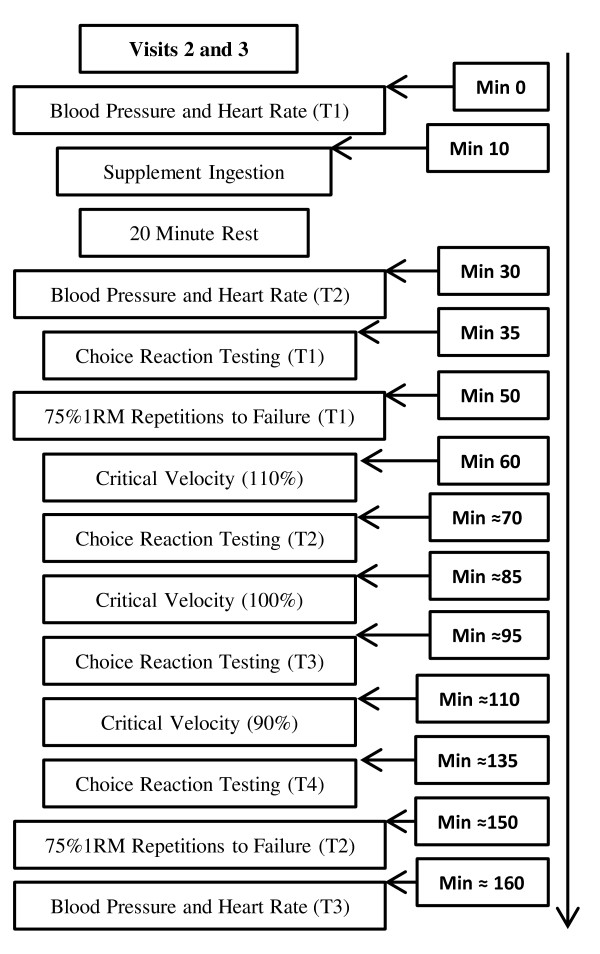
**Study timeline in order from top down with respective time points (T)**.

This study used a randomized, double-blind, placebo-controlled cross-over design. Subjects reported to the human performance laboratory on three separate occasions. All participants completed a baseline/familiarization day of testing, including a maximal graded exercise test for the determination of aerobic capacity (VO_2max_), one-rep maximum (1RM) for bench and leg press to determine 75% of 1RM, choice reaction familiarization, which included single-step visual and audio protocol, one tower protocol, two tower lateral protocol, three tower multi-directional, three tower multi-direction protocol with martial arts stick, and intermittent critical velocity familiarization completed at 110%, 100%, and 90% of VO_2max_. Subjects were instructed to refrain from performing any strenuous activity for 24 h before the subsequent testing sessions (Visit 2, 3). Subjects were also required to maintain their current training program throughout the duration of the study. Additionally, subjects were required to complete a 2-day food log before the second testing day. Two days prior to Visit 3, subjects were asked to consume the exact same foods/beverages eaten and written in their 2-day food log as recorded prior to Visit 2. Subjects reported to the lab in a fasted state (12 h with *ad libitum *water) to ensure that any additional foods/beverages would not influence performance or negate the potential effects of the supplement (SUP) or placebo (PL). Following familiarization, subjects were randomly assigned to ingest either the SUP or PL for Visit 2 and were provided the cross-over treatment on the last testing visit. Twenty minutes after ingestion, blood pressure and heart rate (HR) were re-assessed, and subjects completed a four-question survey (5-point likert scale) that asked them to describe their feelings of energy, fatigue, alertness, and focus at that particular moment [1]. Subjects were asked to rate their energy level, fatigue level, feelings of alertness, and feelings of focus using the following verbal anchors: 1 = very low; 2 = low; 3 = average; 4 = high; 5 = very high. The questionnaire was administered immediately before each choice reaction testing session (Figure 1). Subjects ingested each supplement 20 min prior to testing since that is what is typically practiced by most recreational athletes.

### Choice reaction test

**Figure 2:**
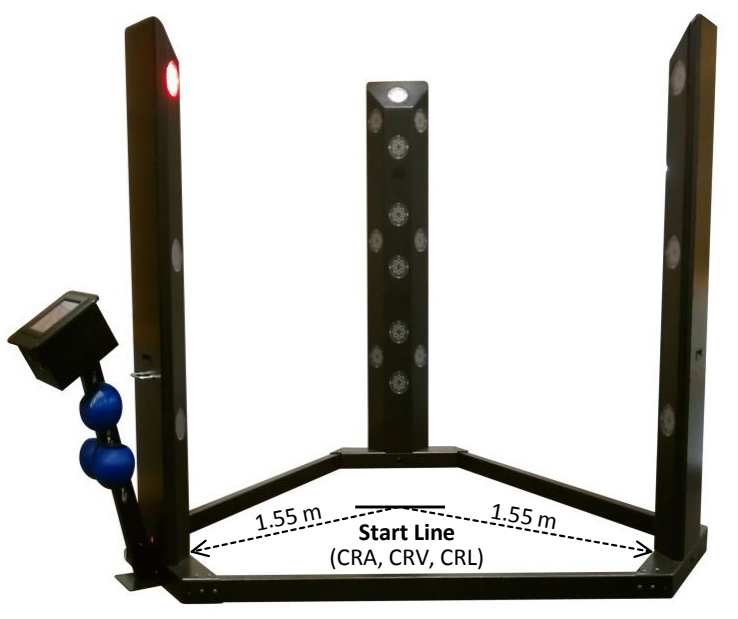
**Makoto II Arena choice reaction time testing equipment with start line identified**. Start line was used for single-step choice reaction with audio and visual cues (CRA, CRV), as well as lateral change of direction (CRL).

Reaction time was measured using the Makoto II Arena testing device (Makoto USA, Centennial, CO, USA). The Makoto II Arena device is constructed in the shape of a triangle that is 2.44 m from base to base and consists of three steel towers that are 1.83 m high with each tower containing 12 targets (Figure 2). Most studies that have investigated choice reaction time failed to utilize a testing device and protocol that is related to sport-specific training and team-sport performance. Due to the fact that most team sports require quick agile movements in different directions, the current investigation sought to utilize a testing device and protocol that measured stationary, lateral, and multi-directional movements. Each subject underwent nine different tests, measuring choice reaction time for each test. First, subjects were tested on a two-tower single step protocol. The reaction test consisted of audio (CRA) and visual (CRV) protocols, each completed separately. The decision to separate reaction time cues was made to accommodate the physiological transduction rate disparities between auditory and visual stimuli [13]. Targets used for this protocol included the middle target on each tower, located approximately at chest-level. The audio protocol consisted of only an auditory stimulus; therefore, a magnetic cover with the label (X) was used to cover the specified target on each tower. The visual protocol consisted of only visual alerts, in which illumination of selected bulbs provided the stimulus. The volume was muted during this test, allowing subjects to use only their visual sense to detect which target illuminated. For each test, subjects stood in the middle of the triangle behind a marked line located 1.55 m from both towers. Subjects stood behind the line with the third target (not being used) directly behind them (Figure 2). Subjects were required to lunge and make contact between their hand and the specified target on either tower. Built-in computer software randomly displayed the target on one of the two towers in the equivalent location.

Reaction time for each subject was calculated using built-in software and was recorded for each trial. Randomization was used to determine whether the subject would complete either the audio or visual protocol first. Subjects repeated each test for a total of three attempts, and the mean of the last two trials was used in analysis. Subjects then completed testing protocols at durations of 15 s, using both audio and visual alerts. Testing procedures included one tower, for which the subjects remained stationary and only used their hands (CRS15), followed by two towers starting from the same line as CRV and CRA, for which subjects moved laterally from side to side (CRL15), and then three towers, for which subjects moved in multiple directions (CRM15); with two attempts for each test. The same testing procedures were followed again; only this time the duration was 30 s (CRS30, CRL30, CRM30). Mean reaction time for each trial was calculated internal to the device and recorded. The average of two back-to-back attempts was calculated and used for analysis. Skill levels indicated how long each target remained illuminated: the lower the level, the longer the target was lit. Each of the aforementioned tests was completed at the first skill level (level 1, 3 s count) to ensure each subject's ability to register a 'hit' with ease. Test-retest reliability of the above protocol calculated from six men at least 2 days apart yielded an ICC > 0.94 and a CV less than 10% for all choice reaction tests. Subjects completed the choice reaction testing protocol with a (three-tower) protocol using a large martial arts stick for two minutes. This protocol was set at the fourth skill level (1.20s count) to mimic the protocol used in a similar study by Hoffman et al. [1]. In our laboratory, test-retest reliability of the above protocol from six men at least 2 days apart yielded an ICC > 0.81. Average reaction time tested at different durations and different types of movements (stationary, lateral, multi-directional) allowed for a comprehensive analysis of agility compared to standard agility tests, such as the pro-shuttle and t-test for which agility speed, and not reaction time, is measured.

### Determination of VO_2max_

All VO_2max _tests were performed on a calibrated treadmill (Woodway Desmo, Waukesha, WI) and measured by indirect calorimetry using a metabolic cart (Parvomedics True One 2300, Sandy, UT, USA). Testing began with a three-minute warm-up stage at 5.15 km/h, while the grade remained at 0% throughout testing. Velocity increased to 10 km/h for the next stage, then increased 2 km/h for every two minutes up to 16 km/h, followed by 1 km/h increments per minute up to 24 km/h. VO_2max _was confirmed if at least two of the following criteria were met: (a) plateau in HR or HR values within 10% of the age-predicted HRmax, (b) plateau in VO_2 _(defined by an increase of no more than 150 ml·min^-1^), (c) respiratory exchange ratio (RER) value greater than 1.15.

### Intermittent critical velocity and intermittent anaerobic running capacity

Intermittent Critical Velocity (iCV) testing protocols were similar to previous studies [2,14]. All iCV tests were performed on a calibrated treadmill (Woodway Desmo, Waukesha, WI, USA). Subjects completed three separate treadmill running tests at intervals of 10 s of running and 10 s of rest at 110%, 100%, and 90% of their peak velocity at VO_2max_. A 25-minute break period was given between running tests. During this break period, subjects performed the choice reaction protocol followed by approximately five minutes of inactivity before the next treadmill test. Each iCV testing session was terminated when the subject could no longer complete a 10s sprint. Distance and total running time were recorded for each participant, and iCV and intermittent anaerobic running capacity (iARC) were calculated using the following equation: (Total Distance = iARC + iCV · total running time). An investigation by Fukuda et al., 2009 [14] examined the reliability of iCV testing in men and women, producing an ICC value of 0.87 and 0.83.

### Muscular strength and endurance

One-repetition maximum bench press and leg press were used to determine the maximum amount of weight a subject could lift one time for each exercise. Subjects performed one warm-up set for bench and leg press (≈50% of estimated 1RM) and rested for two minutes between attempts. Resistance was increased until the participant failed to complete a repetition; subjects performed no more than five attempts. Maximum strength for bench and leg press exercises was measured using plate-loaded iso-lateral bench and leg press (Hammer Strength, Cincinnati, OH, USA). Seat height for bench and leg press was adjusted for each subject and remained consistent throughout the course of the study. A repetition was recorded when subjects lowered the weight in an eccentric contraction and then pushed the weight in a concentric contraction with full joint extension, ensuring the weights made contact with the back bumper pads after eccentric movement. Following 1RM testing, 75% of each subject's 1RM was calculated, and repetitions to fatigue were assessed and recorded at respective weight resistance on subsequent testing visits. Subjects were required to perform a set (at least 5 repetitions) of 75% of their 1RM for familiarization on Visit 1.

### Supplement

**Figure 3:**
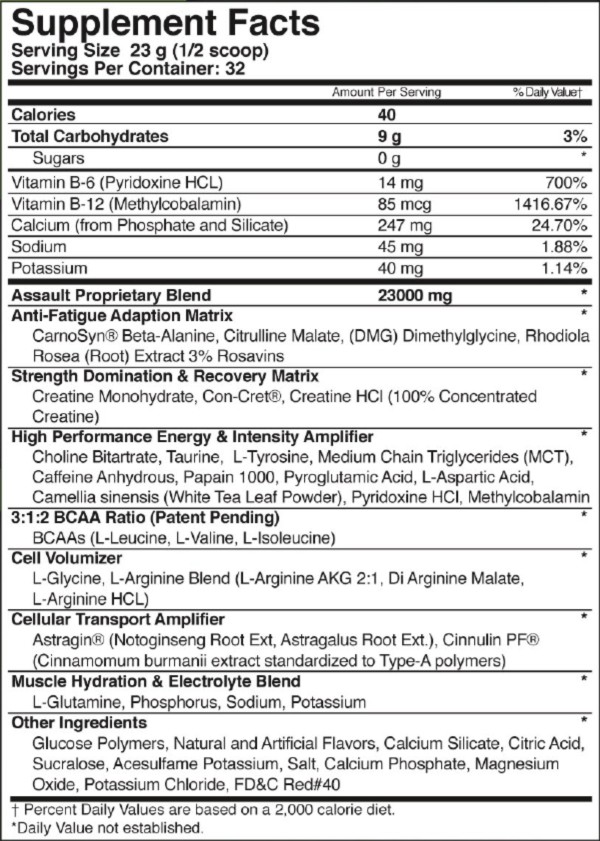
**Dietary supplement (SUP) facts panel**.

On the second and third testing days, subjects consumed 1 scoop (2 servings, 46 g) of either the PL or SUP powder mixed with ≈ 16 oz. of water. The SUP, marketed as Assault™ (MusclePharm, Denver, Colorado, USA), contained BCAAs (6 g), creatine (5 g), beta-alanine (4 g), citruline malate (1.5 g), and caffeine (300 mg). Complete nutritional facts are listed in Figure 3. The PL was flavored maltodextrin, similar in color, taste and flavor to the SUP formulation. An investigator secluded from any data collection prepared and administered both supplement and placebo beverages for all subjects, using the same amount of water and powder for each beverage. Subjects were provided cold water *ad libitum *throughout the study.

### Data analysis

All recurring tests were analyzed using a repeated measure analysis of variance (ANOVA) [time (T1-T4) × drink (SUP vs. PL)]. Significant interactions between time and supplement were analyzed using post-hoc dependent t-tests. In addition, iCV and iARC were analyzed using dependent t-tests. Significant main effects for time and for supplement were analyzed using Bonferroni post-hoc tests to account for multiple comparisons by maintaining family-wise error rates. A p-value less than 0.05 was considered significant for this investigation. All ANOVA assumptions were met, and analyses were performed using the computer program SPSS (PASW Statistics 18.0.0, IBM Corporation, Armonk, NY, USA).

## Results

**Figure 4:**
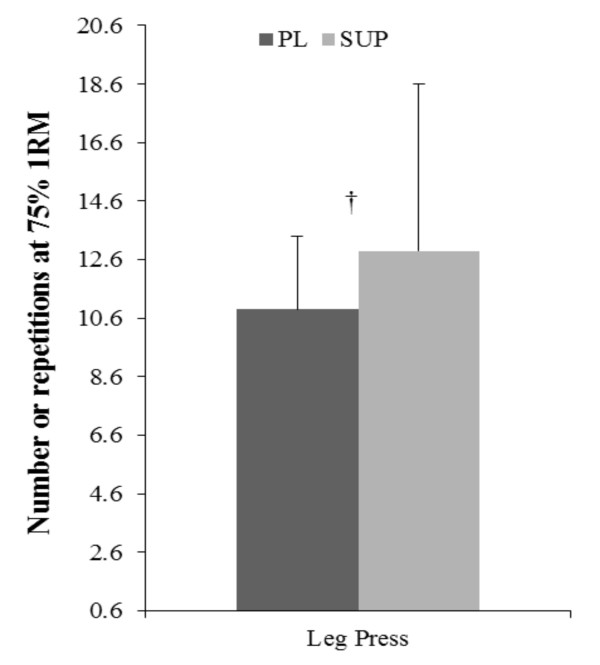
**Total leg press repetitions at 75% of one repetition maximum**. † = Significant difference between SUP and PL, *p *< 0.05. PL = Placebo, SUP = Supplement (Assault™).

**Figure 5:**
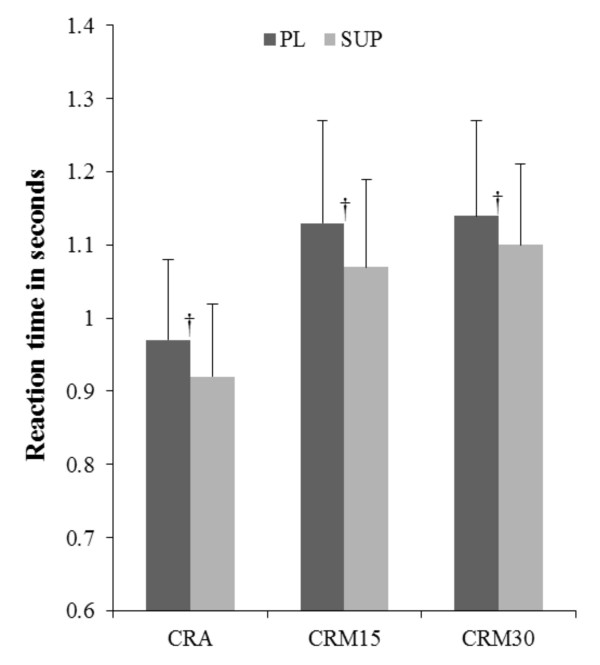
**Mean choice reaction time across all time points**. † = Significant difference between SUP and PL, *p *< 0.05, PL = Placebo, SUP = Supplement (Assault™), CRA = Choice reaction time audio single step, CRM15 = Choice reaction time multi-directional for 15 s, CRM30 = Choice reaction time multi-directional for 30 s.

**Figure 6:**
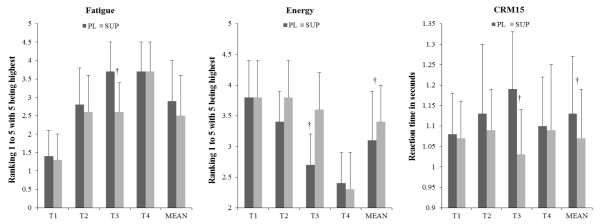
**Fatigue, energy, and choice reaction time comparing means and time points**. † = Significant difference between SUP and PL, *p *< 0.05, PL = Placebo, SUP = Supplement (Assault™), T = time point, CRM15 = Choice reaction time multi-directional for 15 s.

Interactions were observed at time point 3 (T3) for heart rate (HR) (SUP: 102 +/- 12, PL: 94 +/- 15; *p *< 0.05), energy (SUP: 3.6 +/- 0.8, PL: 2.7 +/- 0.5; *p *< 0.05), fatigue (SUP: 2.6 +/- 0.8, PL: 3.7 +/- 0.7; *p *< 0.05) and CRM15 (SUP: 1.03 +/-0.11, PL: 1.19 +/- 0.04; *p *< 0.05. Main effects for time were observed for systolic blood pressure, HR, leg press repetitions, energy, fatigue, alertness, focus, choice reaction visual single step (CRV), choice reaction multi-direction 30 s (CRM30), and stick hits for the two-minute protocol (*p *< 0.05). Post-hoc results for time were independent of the supplement and, therefore, are not reported. Significant main effects for the supplement treatment were observed for leg press repetitions (SUP: 13 +/- 6 reps, PL: 11 +/- 3 reps; *p *< 0.05), energy (SUP: 3.4 +/- 0.9, PL: 3.1 +/- 0.8; *p *< 0.05), alertness (SUP: 4.0 +/- 0.7, PL: 3.6 +/- 0.8; *p *< 0.05), and focus (SUP: 4.1 +/- 0.6, PL: 3.6 +/- 0.8; *p *< 0.05). No significant differences were seen comparing the SUP to PL for iCV (PL: 3.11 +/- 1.2, SUP: 3.15 +/- 1.2 m/s, *p *= 0.95) or iARC (PL: 318.8 +/- 408.7, SUP: 263.3 +/- 416.3 m, *p *= 0.78). Therefore, no significant differences were apparent for total treadmill work between testing days. Results are depicted in Figures 4, 5 and 6 and choice reaction results are presented in Table 1.

**Table 1:** Choice Reaction Time Results

Variable	Treatment	T1	T2	T3	T4	Mean
Single Step Visual (CRV)	PL	0.97 ± 0.14	0.98 ± 0.25	0.94 ± 0.11	0.92 ± 0.15	0.95 ± 0.17
	
	SUP	0.98 ± 0.23	0.93 ± 0.14	0.91 ± 0.16	0.88 ± 0.12	0.93 ± 0.16

Single Step Audio (CRA)	PL	0.98 ± 0.09	0.96 ± 0.13	0.96 ± 0.11	0.95 ± 0.12	0.97 ± 0.11
	
	SUP	0.96 ± 0.10	0.91 ± 0.09	0.89 ± 0.07	0.92 ± 0.11	0.92 ± 0.10^†^

Stationary 15 s (CRS15)	PL	0.54 ± 0.04	0.54 ± 0.07	0.56 ± 0.09	0.55 ± 0.08	0.55 ± 0.07
	
	SUP	0.56 ± 0.06	0.51 ± 0.05	0.51 ± 0.06	0.51 ± 0.06	0.52 ± 0.06

Lateral 15 s (CRL15)	PL	0.91 ± 0.11	0.91 ± 0.15	0.95 ± 0.11	0.89 ± 0.13	0.91 ± 0.13
	
	SUP	0.93 ± 0.11	0.86 ± 0.07	0.88 ± 0.11	0.89 ± 0.12	0.89 ± 0.10

Multi-directional 15 s (CRM15)	PL	1.08 ± 0.10	1.13 ± 0.17	1.19 ± 0.14	1.10 ± 0.12	1.13 ± 0.14
	
	SUP	1.07 ± 0.09	1.09 ± 0.10	1.03 ± 0.11^‡^	1.09 ± 0.16	1.07 ± 0.12^†^

Stationary 30s (CRS30)	PL	0.56 ± 0.07	0.55 ± 0.09	0.54 ± 0.09	0.54 ± 0.07	0.55 ± 0.08
	
	SUP	0.55 ± 0.05	0.52 ± 0.07	0.52 ± 0.06	0.52 ± 0.07	0.53 ± 0.06

Lateral 30s (CRL30)	PL	0.89 ± 0.10	0.93 ± 0.13	0.92 ± 0.10	0.93 ± 0.15	0.92 ± 0.12
	
	SUP	0.91 ± 0.10	0.91 ± 0.11	0.87 ± 0.12	0.90 ± 0.10	0.90 ± 0.11

Multi-directional 30s (CRM30)	PL	1.15 ± 0.13	1.16 ± 0.12	1.11 ± 0.15	1.15 ± 0.12	1.14 ± 0.13
	
	SUP	1.11 ± 0.10	1.10 ± 0.12	1.06 ± 0.12	1.12 ± 0.12	1.10 ± 0.11^†^

Stick Hits (2 min)	PL	63 ± 11	67 ± 10	63 ± 11	61 ± 12	63 ± 11
	
	SUP	66 ± 7	70 ± 10	65 ± 11	65 ± 12	66 ± 10

*PL *placebo; *SUP *supplement; *T *time point; † = significant main effect for supplement, *p *< 0.05; ‡ = significant interaction between time and supplement, *p *< 0.05; All data are reported as mean ± standard deviation.

## Discussion

This investigation was the first to examine the effects of the pre-workout supplement Assault™ on a multi-faceted, exercise testing protocol. The results suggest that the pre-workout supplement significantly improved muscular endurance and choice reaction time.

The primary active ingredient in Assault™ is caffeine. Caffeine is a mild stimulant that affects the central nervous system and has the potential to influence human neuromuscular performance. In an attempt to maximize the effectiveness of caffeine, supplement manufacturers often combine several ingredients, possibly enhancing caffeine's stimulatory potential [1]. A study conducted by Fukuda et al. [15], showed an acute anaerobic effect in men and women with the ingestion of a low dosage of caffeine, BCAAs, and creatine, which could possibly lead to improvements in various aspects of human performance (muscle strength, power, etc).

In the current investigation, a 26.5% reduction in total repetitions (3 reps) at 75% of 1RM was observed from time point 1 to time point 2 in the PL group for leg press, while an 11.7% (2 reps) reduction from time point 1 to time point 2 was observed in the SUP treatment for leg press. Results also indicated positive effects on perceived alertness, energy, and focus, which may have contributed to a 4% improvement in CRA and CRM30, and a 5% improvement in CRM15. These findings are consistent with previous studies [16,17] that also reported significant improvements in choice reaction time following the ingestion of a supplement containing 5-6 mg/kg of caffeine, which is higher than the amount used in the current study (mean = 3.8 +/- 0.7 mg/kg).

There are many possible reasons for the improvements in performance demonstrated in this study. At the cellular level, caffeine enhances neuromuscular transmission and improves skeletal muscle contractility [18]. Green et al. [19] investigated the effects of caffeine (6 mg/kg) on lower body muscular endurance with repetitions performed to volitional failure. Results indicated that caffeine was associated with significantly higher repetitions (12.5 ± 4.2, 9.9 ± 2.6) during the third set of leg press exercises compared to PL [19]. In agreement, the current investigation showed significant increases in both total leg press repetitions (SUP 13 ± 6, PL 11 ± 3, *p *= 0.021) at T1 and T2 (SUP 12 ± 5, PL 8 ± 6, *p *= 0.024), although no significant time and SUP interaction was found (Figure 4).

A study by Barry et al. [20] examining caffeine-induced arousal effects on performance and auditory event-related potentials in the brain, indicated a reduction in reaction time and suggested that caffeine differentially improves processing aspects related to task performance [20]. The decrease in CRA times may have had an effect on the improvement in CRM15 and CRM30 because they also include an auditory component. Figure 5 illustrates that the reduction in multi-directional choice reaction time (CRM) for 15 s and 30 s was most likely due to the significant decrease in choice reaction time using an auditory stimulus (CRM15, SUP: 1.07 ± 0.12 s, PL: 1.13 ± 0.14 s, *p *= 0.007; CRM30, SUP: 1.10 ± 0.11 s, PL: 1.14 ± 0.13 s, *p *= 0.013). Caffeine's stimulatory effects on the central nervous system seem to manifest in performance in activities which require quick reactions and movements [18]. Thus, it is evident that choice reaction time improved following the ingestion of 3.8 +/- 0.7 mg/kg of caffeine (approx. 300 mg) for recreationally-trained males between the ages of 18 and 35 years. It remains unknown as to why the single-tower and two-tower reaction times were not significantly different between groups, as they included both audio and visual cues. It is hypothesized that the three-tower, multi-directional reaction time tests required the greatest movement and consequently the greatest task difficulty and overall mental and physical demand; thus, fatigue played a larger role in reaction time compared to the single- and two -tower tests. Independent of fatigue, caffeine could have influenced the multi-directional reaction tests because of the increased task complexity. Therefore, the improvements in the three-tower, multi-directional reaction time protocol after supplementation could have been attributed to both a reduction in fatigue as well as enhanced cognitive function. More research is required to determine the influence of the SUP used in the current investigation on reaction times using audio versus visual cues and various sport-specific movements.

An investigation by Fukuda et al. [15] examined whether a pre-workout supplement with similar ingredients (caffeine, creatine, amino acids) to the SUP used in the present study would impact anaerobic running capacity and critical velocity. Results showed no significant different between the SUP and the PL for critical velocity. However, it was reported that the SUP significantly increased anaerobic running capacity compared to the PL. In the present study, no significant differences were seen in anaerobic running capacity or intermittent critical velocity with the ingestion of the pre-workout supplement. However, the study by Fukuda et al. [15] was conducted in college-aged men and women and indicated an overall increase in anaerobic running capacity of 10.8% compared to the placebo with an increase in time to exhaustion at 110%, 105%, and 100% of peak velocity. Suggesting, the multi-ingredient supplement used in the investigation had effects on anaerobic performance only, which is similar to the current findings. Differences between findings could be related to the subjects and protocols used in the investigations. More research is needed regarding continuous anaerobic running capacity, critical velocity, and pre-workout supplements containing similar ingredients to those found in the SUP.

The SUP treatment decreased fatigue, increased energy, and improved choice reaction time (CRM15). These data suggest that at time point three (T3), around 95 min into the exercise protocol, as the subjects began to fatigue, the SUP caused a delay in fatigue and an increase in energy as evidenced by significantly faster choice reaction times (CRM15). Therefore, it appears that a single scoop (2 doses) of the SUP taken 20 min prior to exercise or training could allow for more total work with a higher quality (faster reactions) of work. Additionally, across all choice reaction time tests, mean values were faster for the SUP compared to the PL during time points two, three, and four with the exception of CRL15 at time point four, where the means were the same. At time point one; 20 min after ingestion, before any other exercises were completed, the SUP produced faster reactions in only four of the eight reaction tests. These findings suggest that the SUP began to effect performance only after the first round of testing, suggesting the largest factor related to improved performance was the ability of the SUP to delay fatigue and maintain high levels of energy.

Some limitations of this investigation were the use of lower percentages of speed at VO_2max _to measure iCV and iARC compared to other investigations [14], which could have reduced the ability to find significant changes in anaerobic running performance, although it is more likely that a single dose of the SUP had little or no effect on iCV or iARC because creatine and beta alanine both require loading or prolonged use to see performance benefits, particularly in iCV or iARC.

Also, the fact that the SUP had multiple ingredients makes it impossible to identify which specific ingredients contributed to improved performance. Beta-alanine and creatine, two of the active ingredients in the pre-workout supplement (MusclePharm Assault™) have both been shown to improve anaerobic capacity [8,21]. Most studies that investigated the effects of beta-alanine on exercise performance reported an increase in muscle carnosine [9,21,22]; although, those studies incorporated beta-alanine loading, stating that a longer supplementation period (≥ 4 weeks) could produce greater increases in muscle carnosine. In theory, a significant increase in muscle carnosine could result in an increase in muscle buffering capacity [22], translating to improvements in anaerobic running capacities by decreasing the accumulation of hydrogen ions. In the present study, no significant differences were seen in anaerobic running capacity and intermittent critical velocity with the one-time ingestion of the pre-workout supplement. Creatine and beta-alanine both require loading periods of several weeks to positively influence exercise performance. As a result, the only active ingredients likely to influence performance in this investigation were caffeine and B-vitamins.

Study limitations also relate to the characteristics of the subjects who participated in the current investigation. While the men were recreationally-trained with strength values in the 75^th ^and 90^th ^percentile (ACSM guidelines) for bench and leg press 1RM and VO_2max _values in the 60^th ^percentile, the SUP may not have the same effects in men who are more anaerobically- or aerobically-trained. Nonetheless, the subjects in the current study should represent a large population of active men who regularly exercise. In addition, two-day food logs indicated subjects consumed an average of 31.5 +/- 109.4 mg of caffeine per day in their normal diet, and active men who regularly consume larger quantities of caffeine may not experience the same results as the men in the current investigation.

## Conclusion

The overall findings of this investigation indicate that the supplement Assault™ can significantly improve perceived feelings of energy, focus, and alertness leading to a significant improvement in multi-directional choice reaction time and single-step choice reaction time with an auditory stimulus. Results also support that the SUP may reduce both feelings of fatigue and actual fatigue, which can result in performance increases in both muscular endurance and reaction time. Additionally, from a practical perspective, college-age males who recreationally participate in sports where auditory stimuli are abundant, such as team sports where verbal cues are encouraged may experience increases in performance after ingesting the pre-workout supplement used in the current study (Assault™).

## Competing interests

All authors have no financial interests concerning the outcome of this investigation. This publication should not be viewed as endorsement by the investigators, the United States Sports Academy, the University of Oklahoma, or the Journal of the Nutrition & Metabolism.

## Authors' contributions

BS, KC, CT, KK, DF, EE, and JM participated in the study design and helped draft the manuscript while aiding in data analysis. BS, KC, CT, and EE participated in data collection. Additionally, all authors read and approved the final manuscript.
